# What kinds of speeches motivate climate action?

**DOI:** 10.1098/rsos.241563

**Published:** 2025-04-30

**Authors:** Lukas Reinhardt, Harvey Whitehouse

**Affiliations:** ^1^ Centre for the Study of Social Cohesion, University of Oxford, Oxford, UK; ^2^ Identity and Conflict Lab, Yale University, New Haven, CT, USA

**Keywords:** climate action, humanity, fusion, identity

## Abstract

The scientific evidence on the harmful consequences of climate change is clear yet appeals to scientific evidence alone may not be enough to inspire sufficient climate action. We analysed the effectiveness of four different speeches in video format delivered by a Global South politician to strengthen psychological bonding with humanity at large and motivate climate action in the form of donations with samples from the US, UK and South Africa. Each speech focused on one core argument: scientific evidence, morality, globally shared life experiences or humanity’s shared ancestry. All speeches significantly increased bonding with humanity and donations. On average, the speeches were equally effective at increasing donations. The speech that appealed to humanity’s shared ancestry had a stronger effect on bonding with humanity than the other three speeches and the speeches that appealed to globally shared life experiences and humanity’s shared ancestry made participants feel less sad, angry and helpless than the speeches that appealed to scientific evidence and morality. We also present evidence on the effects of the speeches delivered by a Western academic in the UK and we discuss implications of our findings for future research and practical efforts to motivate climate action.

## 1. Introduction

Limiting global warming to 1.5°C or 2°C requires urgent action on a global scale [1]. Although the scientific arguments are clear and compelling, the world community is unlikely to meet the challenge without a rapid increase in commitment and behaviour change (both structural and at the level of grassroots action and consumption patterns). Part of the problem is to provide politicians with narratives capable of increasing and spreading commitment to addressing the climate crisis. However, as long as people do not sufficiently care about the fate of humanity at large, it might be challenging to overcome the incentive structure of global collective action problems. In this article, we analysed the effectiveness of different speeches to foster bonding with humanity and motivate action against climate change.

Previous research suggests that religious, national and political identities can be harnessed to motivate climate action [2,3].^1^ It has also been argued that fostering global identity can motivate pro-environmental action [9–11].^2^ However, a key challenge in fostering global identity is to find ways to make global identity sufficiently strong to motivate effective prosocial action at scale. The most potent from of extreme group cohesion known to social psychology is ‘identity fusion’—a visceral sense of oneness capable of motivating extreme pro-group action [17–19]. Identity fusion emerges as a consequence of perceptions of shared essence with a group that makes personal identity and group identity ‘fuse’ together—hence the term identity *fusion*. For an individual who is fused with a group, any threat to the group is taken personally leading to a willingness to defend the group against the threat even at high personal cost.^3^

There are two known pathways to identity fusion: perceptions of shared transformative experiences (e.g. [20]) and perceptions of shared biological kinship (e.g. [22]). In the case of shared transformative experiences, perceptions of shared essence with the group emerge if emotional events that are stored in episodic memory and perceived as shared with a group lead to reflection on their meaning (enhanced by causal opacity) and subsequent personal identity transformation. In the case of perceptions of shared biological kinship, perceptions of shared essence can be caused by phenotypic matching but also by narratives of shared ancestry on the tribal, ethnic or national level [23]. The identity fusion literature distinguishes ‘local’ and ‘extended’ fusion [24]. Local fusion can emerge within relatively small groups that are characterized by direct personal contact such as families, small tribes or units of fighters whereas extended fusion can occur in larger groups in which most group members do not have direct personal contact such as ethnic groups, nations or religious communities. Reinhardt & Whitehouse [25] present evidence that extended fusion can even occur on a global level with humanity at large as the target group and motivate prosocial action on a global scale.^4^

While social identification [27,28] may often be strongly correlated with identity fusion, these contrasting forms of group alignment are established through empirically distinguishable pathways. Identity fusion requires perceived sharedness of features that are essential to one’s personal identity, whereas identification emerges if prototypical features that are not essential to one’s personal identity are perceived as shared with the group [17,24,29]. A key difference between both forms of group cohesion is that identification can be characterized by a hydraulic relationship between group and personal identities—making one identity salient makes the other less salient—whereas fusion can be characterized by a synergistic relationship—one identity activates the other [18]. Although we do not focus on theoretical or empirical differences between social identification and identity fusion in this article, many studies have demonstrated that identity fusion is more effective than social identification in producing extreme forms of pro-group action and group loyalty [17,18,23,24,30].^5^

In this article, we explored whether the two pathways to identity fusion can be harnessed to strengthen bonding with humanity and motivate climate action by analysing the effects of appeals to globally shared transformative life experiences and global biological kinship that makes all humans part of a single human family. We benchmarked these two strategies with appeals to scientific evidence and a moral argument that highlighted the injustice of climate change—two communicative strategies frequently used in the public debate on climate change. Due to the extreme bonding power of shared transformative experiences and shared biology, we hypothesized that the shared experiences speech and the shared biology speech would have a stronger effect on fusion with humanity and donations than the science and morality speeches.

In order to compare the effectiveness of these four appeals ((i) scientific evidence, (ii) morality, (iii) globally shared experiences and (iv) globally shared biology), we ran a pre-registered online experiment with samples from the US, UK and South Africa. We recorded four videos that we used as treatments in a between-subjects design. Each video included a speech delivered by Ralph Regenvanu—the former Minister for Foreign Affairs of the Republic of Vanuatu and at the time of data collection Vanuatu’s Minister for Climate Change—for more ambitious climate action. The beginning and end of the speeches were identical. However, the middle part varied to include one of the arguments listed above. In the experiment, we had four conditions in which we exposed participants to one of the speeches before measuring fusion with humanity and donations to a climate charity and one control condition in which we measured these outcomes *before* exposing the participants to a speech.

We found that all speeches significantly increased fusion with humanity and donations for the climate charity compared to the control condition. The shared biology speech resulted in significantly higher levels of fusion with humanity than in the other three speeches while there were no significant differences with regard to fusion with humanity between the science, morality and shared experiences speeches. The four speeches were equally effective at increasing donations for the climate charity. Furthermore, the two speeches that were motivated by identity fusion theory—the shared experiences speech and shared biology speech—evoked significantly fewer negative emotions (sadness, anger, helplessness) than the science and morality speeches.

We compared the data from the UK with an earlier version of the study in the UK in which the speeches were delivered by a Western academic (second author Whitehouse) rather than by a Global South politician. In contrast with the study with the speeches delivered by the Global South politician, the science speech and the shared biology speech did not significantly increase fusion with humanity and donations compared to the control condition in the study with the speeches delivered by the Western academic. However, in a difference in difference analysis with both datasets, only the interaction between the shared biology speech (versus control condition) and the speaker (Global South politician versus Western academic) on fusion with humanity was significant. Perhaps the globally shared biology argument was perceived as more salient or authentic by UK participants when coming from a Global South politician rather than from a Western academic. Moreover, in the study with the speeches delivered by the Western academic, the shared experiences speech and shared biology speech also evoked significantly fewer negative emotions than the science and morality speech.

## 2. Experimental design, data and hypotheses

In our between-subjects experiment, we analysed the effectiveness of four short speeches to motivate climate action. The speeches were structured similarly and used the same opening and closing paragraphs but differed in the core argument they used in the middle paragraph to motivate climate action. Speech 1 appealed to scientific evidence, speech 2 made a moral argument by appealing to the injustice that poor countries suffer the most from climate change, speech 3 appealed to the fact that all humans share similar life experiences and speech 4 appealed to the fact that all humans share common ancestors and thus are all members of a great global family. We recorded a version of these speeches delivered by Ralph Regenvanu—the former Minister for Foreign Affairs of the Republic of Vanuatu and at the time of data collection Vanuatu’s Minister for Climate Change—and exposed participants of the experiment to the resulting videos as treatments in the experiment. Since Mr Regenvanu agreed to record all four speeches in the same room, features such as video background, lighting, camera angle, tone of voice, volume, etc. were constant across videos such that the only feature that varied between videos was the central argument made in the middle paragraph of the speech. The scripts of the speeches were as follows.^6^

*Opening paragraph (identical across speeches)*: ‘It is a sad reality that most countries don’t do enough to fight climate change but failure to act is going to cost us all. If each and every one of us around the world were to act with urgency right now, we could make huge changes overnight—but what would make us do that?’

*Middle paragraph science speech*: ‘To make us act as one we need to remember that human activities are causing greenhouse gases which are in turn making the planet heat up. Extreme weather events are becoming more frequent in every region of the world and the scientific evidence that we need to limit global warming to 1.5 degree Celsius is clear.’

*Middle paragraph morality speech*: ‘To make us act as one we need to remember that the rich countries have contributed the most to climate change and built their wealth on the use of fossil fuels while the poorest and most vulnerable countries who have contributed the least to climate change suffer the most from its consequences. An example is the island nation of Vanuatu which was directly hit by two major cyclones in rapid succession this year, for the first time in living memory.’

*Middle paragraph shared experiences speech*: ‘To make us act as one we need to think as one. Just reflect for a moment on any experience that has made you who you are today. Maybe it would be the birth or the death of a person dear to you or maybe some other event. Whatever it is, you can be sure that other people far away on the other side of the world have been through something incredibly similar.’

*Middle paragraph shared biology speech*: ‘To make us act as one we need to remember that we are all members of one great human family. We all descend from a common human ancestor and so we are all relatives in a real biological sense. Since we are all members of the same great human family, we must think and act accordingly.’

*Closing paragraph (identical across speeches)*: ‘If we can act together we can collectively reduce our carbon footprint. And if we can do that, we can save each other and our planet.’

We recruited participants from the US, the UK and South Africa via the online survey platform Prolific, thus covering participants from three continents, the Global South and the Global North. Our selection of countries also allowed us to recruit participants who were fluent in English—our second screening criteria besides nationality—to make sure that we could expose all participants to the same speeches. From each country, we had 1000 participants. In the following, we present the design of our between-subjects experiment in chronological order. First, we measured demographics and conducted an attention check. Then, participants were randomly assigned to one of five conditions: science, morality, shared experiences, shared biology and control. Participants in the first four conditions were exposed to the respective video before we measured outcomes. In the control condition, we measured outcomes first before we showed the participants one of the four videos.

The first outcome was fusion with humanity at large, measured using a pictorial scale originally proposed by Swann *et al*. [33]. We coded fusion with humanity from 1 to 5 where higher values were associated with higher fusion levels. The same measurement method for fusion with humanity was used by Reinhardt & Whitehouse [25]. The second outcome was a donation decision where participants were informed that they automatically entered a lottery to win £10 and that they had to decide how much to keep for themselves and how much to donate to The Gold Standard, a climate action charity, if they were to win the lottery. Determining actual payouts by lottery is a cost-efficient incentivization approach frequently used in behavioural economics [34]. We randomly selected 25 participants and allocated the money as these participants indicated. We also measured how sad, angry, helpless and empowered the participants felt on a scale from 1 to 5. These emotions play a role in real-life climate debates and while much of the complex interplay between emotions and climate action still needs to be explored, positive and negative emotions can promote climate action [35,36]. After exposure to the video and outcome measurement, we included some post-treatment measures, asked a comprehension check question about the content of the speech and asked whether participants faced any technical problems.

We pre-registered the main hypothesis that the shared experiences speech and the shared biology speech would be more effective at fostering fusion with humanity and motivating donations than the science and morality speech.^7^ We also pre-registered that we would analyse potential differences in emotions across treatment, and in particular seek to establish whether the shared experiences speech and the shared biology speech would generate higher levels of empowerment and lower levels of helplessness than the science and morality speech. For the analyses we used OLS regressions. We have not pre-registered and we did not perform multiple comparison corrections.

As pre-registered, we excluded participants who failed the attention check, reported technical problems or failed the comprehension check leaving us with a final sample size of 2705 participants. The mean age was 37, 55% of the participants were female and 63% had a college degree. Forty-four per cent indicated that their income lay in the lowest third of the population, 49% indicated that their income lay in the middle third of the population and 7% indicated that their income lay in the highest third of the population. Electronic supplementary material, table S1 provides descriptives for each country. The data collection took place on 6−7 September 2023. We paid participants £0.60 as a baseline payment, which resulted in average hourly rates of £9.04 for US participants, £5.43 for South African participants and £9.43 for UK participants. The differences in average hourly wages occurred due to differences in completion times across countries. Based on earlier rounds of data collection in the UK (see §4), we chose the baseline payment that would result in an expected average hourly rate of £9.00, which was assessed by Prolific as ‘good’.

## 3. Results

We started by investigating whether fusion with humanity predicted donations in the absence of any treatment in an exploratory manner. In the control condition, both variables were correlated (*r* = 0.143, *p* < 0.001, see electronic supplementary material, table S2) and their correlation was higher than the correlation between donations and having a college education (*r* = 0.046, *p* = 0.285), right-wing orientation on the political left-right spectrum (*r* = −0.061, *p* = 0.160) and religiosity (*r* = 0.084, *p* = 0.052). In the control condition, participants who had the lowest possible value on the fusion with humanity scale of 1 donated on average £2.08 while participants with the highest possible value on the fusion with humanity scale of 5 donated on average £3.44.^8^

**Figure 1.**
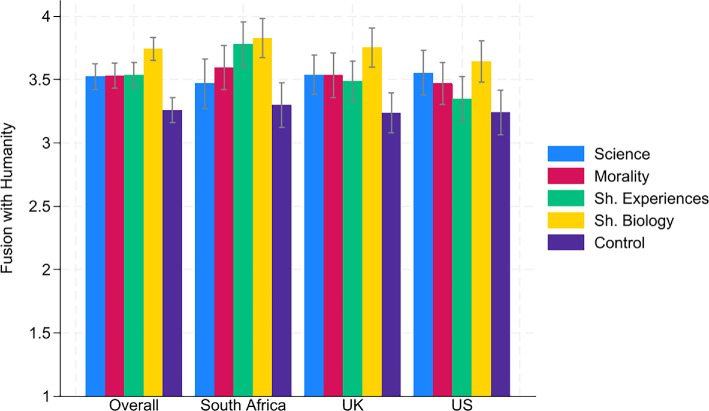
Fusion levels. *Notes*: Confidence intervals have a confidence level of 0.95. The fusion scale ranged from 1 (weakest fusion level) to 5 (strongest fusion level).

**Figure 2.**
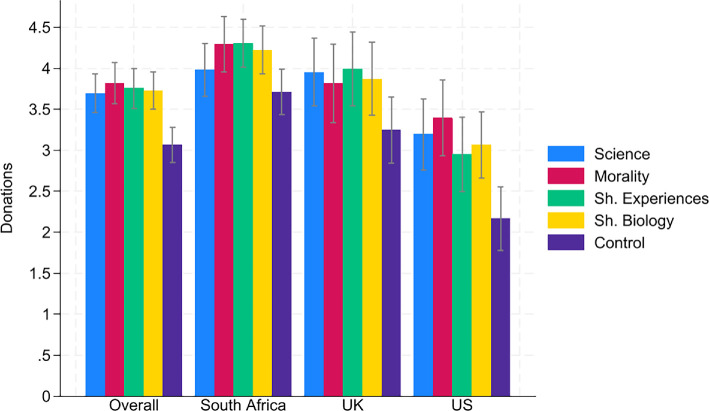
Donations. *Notes:* Confidence intervals have a confidence level of 0.95. Participants could donate any amount between £0 and £10.

Next, we turned to the treatment effects on fusion with humanity and donations. Figure 1 shows fusion with humanity in the five conditions for all three countries. Overall, all speeches significantly increased fusion with humanity compared with the control condition (all *p*-values < 0.001, OLS with robust s.e., see electronic supplementary material, table S4)^9^ and the shared biology speech had a stronger impact on fusion with humanity than the other three speeches (all *p*-values < 0.004, OLS with robust s.e., see electronic supplementary material, table S6).^10^ There were no significant differences between the science, morality and shared experience speech. Figure 2 presents donations in the five conditions for all three countries. As in the case of fusion, all four speeches increased donations for the climate charity (all *p*-values < 0.001, OLS with robust s.e., see electronic supplementary material, table S5).^11^ However, there were no significant differences between the four speeches.^12^

**Figure 3.**
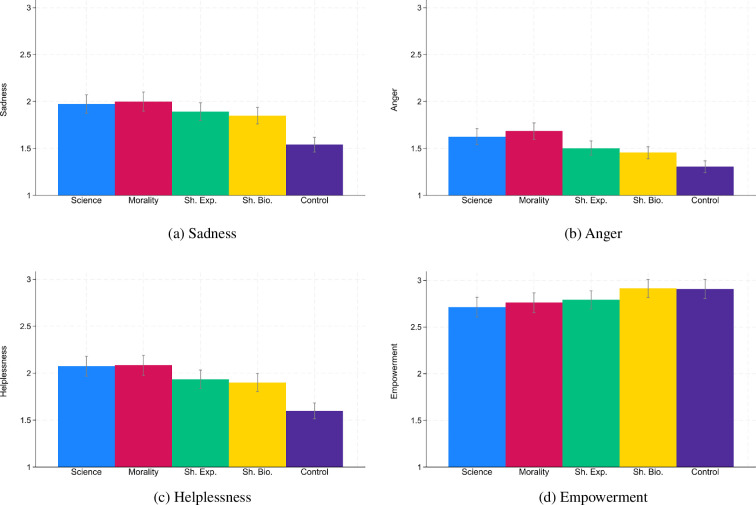
Emotions across conditions. *Notes:* Confidence intervals have a confidence level of 0.95. Emotions were measured from 1 (not feeling this emotion at all) to 5 (feeling this emotion strongly).

Figure 3 presents emotions across all five conditions. We started by testing our pre-registered hypotheses that the two treatments that were informed by fusion theory—shared experiences and shared biology—would generate higher levels of empowerment and lower levels of helplessness than the science and morality treatment. With regard to helplessness, the *p*-values of the differences between shared experiences and the two other speeches oscillated around 0.05 (comparison shared experiences and science: *b* = −0.14, *p* = 0.058; comparison shared experiences and morality: *b* = −0.15, *p* = 0.045, OLS with robust s.e., see electronic supplementary material, table S7) and the *p*-values of the differences between shared biology and the other two speeches were smaller than 0.05 (comparison shared biology and science: *b* = −0.17, *p* = 0.017; comparison shared biology and morality: *b* = −0.18, *p* = 0.013, OLS with robust s.e.). With regard to empowerment, there were significant differences for the shared biology speech (comparison shared biology and science: *b* = 0.20, *p* = 0.007; comparison shared biology and morality: *b* = 0.15, *p* = 0.041, OLS with robust s.e., see electronic supplementary material, table S8), but not for the shared experience speech (comparison shared experiences and science: *b* = 0.08, *p* = 0.290; comparison shared experiences and morality: *b* = 0.03, *p* = 0.690, OLS with robust s.e.).

Next, we grouped the two treatments that were informed by fusion theory and appeal to shared humanity together in an exploratory manner. We compared these sharedness speeches (shared experiences and shared biology) with the non-sharedness speeches (science and morality) and we found significant effects on all four emotions using OLS regressions with robust s.e. and a ‘sharedness’ dummy variable (excluding control group observations). Sharedness speeches made participants feel less sad (*b* = −0.12, *p* = 0.015, see electronic supplementary material, table S9), angry (*b* = −0.18, *p* < 0.001) and helpless (b = −0.16, *p* = 0.002) and more empowered (*b* = 0.12, *p* = 0.027).

## 4. Varying the speaker

### 4.1. Experimental design, data and hypotheses

Prior to the data collection with the videos recorded by Ralph Regenvanu discussed in §§2 and 3, we collected data using the same speeches recorded by author Harvey Whitehouse with a UK sample.^13^

The design was similar to the one discussed in §2 with the following differences. First, we included a petition where participants were asked whether they think that the UK government should engage in more ambitious action against climate change as an additional outcome. We dropped this in the data collection with Ralph Regenvanu’s speeches because we observed high shares of participants willing to sign but very similar rates across treatment from which we concluded that our speeches were unlikely to shape deeply held policy beliefs that affect whether or not participants would sign the petition. Second, we measured emotions in the control group after we showed one of the videos instead of before exposure to the video as we did in the data collection with Ralph Regenvanu’s speech. We decided to measure emotions in the control conditions before exposure to the video with Ralph Regenvanu’s speeches to investigate how exposure to the speeches would affect emotions compared with the control condition.

We collected data at Prolific and after excluding participants according to the same pre-registered criteria as in the data collection with Ralph Regenvanu’s speeches, we ended up with 928 participants who were UK citizens. The mean age was 42 and the median income was in the range of 20 000−39 999 pounds per year. Female participants comprised 59% and male 41%. The highest degree was a high school degree for 34% of the participants, a bachelor’s degree for 46% of participants, a master’s degree for 15% of the participants and another degree or no degree for the remaining participants. We excluded all participants who took the study with Harvey Whitehouse’s speeches from the participant pool for the study with Ralph Regenvanu’s speeches, so no participants participated in more than one study. The data collection took place on 14 July 2023. We paid participants £0.60 as a baseline payment, which resulted in an average hourly rate of £8.34.

We pre-registered the main hypotheses that the shared experiences speech and the shared biology speech would be more effective at fostering (i) fusion with humanity, (ii) donations, (iii) the willingness to support the petition (iv) and the share of participants who donated the highest possible amount. We also pre-registered that we would explore differences in emotions between treatments, and in particular, whether the two treatments that were informed by fusion theory (shared experiences and shared biology) would generate higher levels of empowerment and lower levels of helplessness than the science and morality treatment.^14^

### 4.2. Results

First, we compared fusion levels and donation decisions in the data collections with both speakers. For the following comparisons, we will only include the UK participants from the data collection with Ralph Regenvanu. We did not pre-register any comparisons between the two datasets, so any comparative analyses were exploratory. We will turn to the analyses of the pre-registered hypotheses about comparisons between speeches of the Western academic at the end of this subsection.

**Figure 4.**
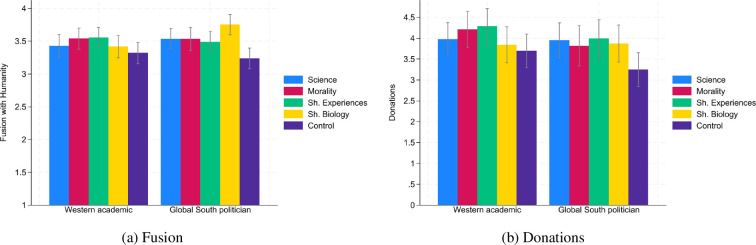
Comparison between speakers (UK participants only). *Notes:* Confidence intervals have a confidence level of 0.95. The fusion scale ranged from 1 (weakest fusion level) to 5 (strongest fusion level). Participants could donate any amount between £0 and £10. The bars for the Global South politicians only include UK participants.

Figure 4 presents fusion with humanity and donations across conditions for both speakers. Tables 1 and 2 show treatment effects on fusion with humanity and donations relative to the control condition (electronic supplementary material, tables S11 and S12 present the results with control variables). For the Global South politician all effects on fusion with humanity had *p*-values < 0.029 (columns 5−8 in table 1, OLS with robust s.e.) and all effects on donations had *p*-values < 0.075 (columns 5−8 in table 2, OLS with robust s.e.).^15^ For the Western academic, the effects of the science speech and the shared biology speech on both outcomes have completely lost significance (with *p*-values between 0.334 and 0.628, columns 1 and 4 in tables 1 and 2, OLS with robust s.e.) while the effects of the morality speech and the shared experiences speech on both outcomes were comparable to the effects of these two speeches delivered by the Global South politician with regard to effects sizes and *p*-values (Western academic: *p*-values ranging between 0.035 and 0.087, columns 2 and 3 of tables 1 and 2; Global South politician: *p*-values ranging between 0.015 and 0.075, columns 6 and 7 of tables 1 and 2, OLS with robust s.e.).^16^

**Table 1.** Effects on fusion with humanity for both speakers (UK participants only). *Notes:* OLS regressions with robust s.e. The fusion scale ranged from 1 (weakest fusion level) to 5 (strongest fusion level). The independent variables are dummies that equal 1 if the participant was assigned to the respective condition. All columns only include participants from the control condition and the condition that relates to the respective dummy variable, e.g. column (1) only includes participants from the science and control conditions. Columns (1), (2), (3) and (4) describe the dataset with the speeches from the Western academic. Columns (5), (6), (7) and (8) include the UK participants from the dataset with the speeches from the Global South politician.

	Western academic				Global South politician			
	(1) fusion	(2) fusion	(3) fusion	(4) fusion	(5) fusion	(6) fusion	(7) fusion	(8) fusion
Science	0.110				0.299***			
	(0.370)				(0.008)			
Morality		0.222*				0.294**		
		(0.057)				(0.015)		
ShExp			0.239**				0.249**	
			(0.035)				(0.028)	
ShBio				0.0975				0.513***
				(0.420)				(0.000)
_cons	3.319***	3.319***	3.319***	3.319***	3.238***	3.238***	3.238***	3.238***
	(0.000)	(0.000)	(0.000)	(0.000)	(0.000)	(0.000)	(0.000)	(0.000)
*N*	371	367	381	355	377	343	388	378
*R* ^*2*^	0.002	0.010	0.012	0.002	0.018	0.017	0.012	0.052

*p*-values in parentheses

* p < 0.1, ** *p* < 0.05, *** *p* < 0.01

**Table 2.** Effects on donations for both speakers (UK participants only). Notes**:** OLS regressions with robust s.e. Participants could donate any amount between £0 and £10. The independent variables are dummies that equal 1 if the participant was assigned to the respective condition. All columns only include participants from the control condition and the condition that relates to the respective dummy variable, e.g. column (1) only includes participants from the science and control conditions. Columns (1), (2), (3) and (4) describe the dataset with the speeches from the Western academic. Columns (5), (6), (7) and (8) include the UK participants from the dataset with the speeches from the Global South politician.

	Western academic				Global South politician			
	(1) donation	(2) donation	(3) donation	(4) donation	(5) donation	(6) donation	(7) donation	(8) donation
Science	0.281				0.706**			
	(0.334)				(0.017)			
Morality		0.518*				0.572*		
		(0.087)				(0.075)		
ShExp			0.586*				0.746**	
			(0.053)				(0.017)	
ShBio				0.146				0.624**
				(0.628)				(0.043)
_cons	3.695***	3.695***	3.695***	3.695***	3.246***	3.246***	3.246***	3.246***
	(0.000)	(0.000)	(0.000)	(0.000)	(0.000)	(0.000)	(0.000)	(0.000)
*N*	371	367	381	355	377	343	388	378
R^2^	0.003	0.008	0.010	0.001	0.015	0.009	0.015	0.011

*p*-values in parentheses

* p < 0.1, ** *p* < 0.05, *** *p* < 0.01

However, the interactions between the respective treatment dummies and a speaker dummy on fusion with humanity and donations were not significant with the exception of the interaction of the shared biology treatment (versus control condition) with the speaker dummy (Global South politician versus Western academic) on fusion with humanity. In this case the effect of the shared biology speech was 0.42 fusion scale points stronger (the fusion scale ranges from 1 to 5) if the speech was delivered by the Global South politician rather than by the Western academic (*p* = 0.012, OLS with robust s.e., see electronic supplementary material, table S14). Hearing an appeal from a person from the Global South that all humans belong to a great family made the notion of a global family perhaps less abstract to the British participants than hearing the same appeal from a fellow British citizen.

**Figure 5.**
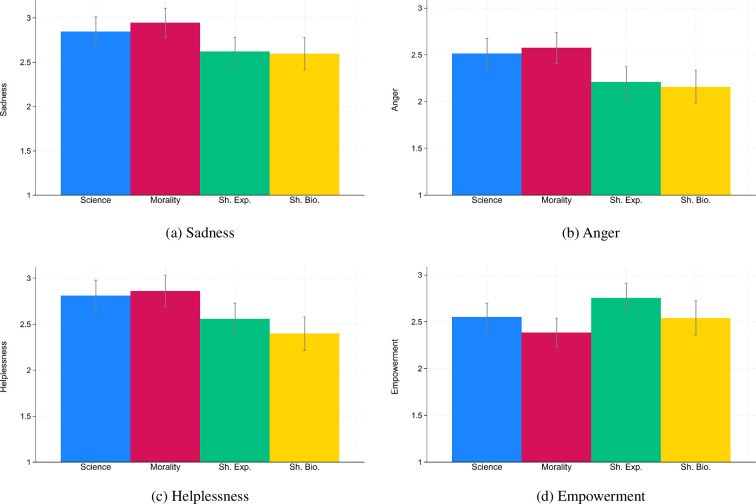
Emotions in the data collection with the Western academic. *Notes:* Confidence intervals have a confidence level of 0.95. We also asked participants in the control conditions which of these emotions the video evoked in them. Control group participants were not included in this figure though.

Turning to the pre-registered analyses, the differences between speeches in the data collection with the speech delivered by the Western academic were not significant. Moreover, we did not find significant differences between speeches with regard to the share of participants who donated the maximum amount and the share of participants who were willing to sign a petition for more climate action (see electronic supplementary material, figures S1, S2). With regard to emotions, we found similar patterns as in §3 (see figure 5). The two speeches that appealed to sharedness made participants feel less sad (*b* = −0.29, *p* = 0.001, OLS with robust s.e., see electronic supplementary material, table S16), angry (*b* = −0.38, *p* < 0.001) and helpless (*b* = −0.35, *p* < 0.001) and more empowered (*b* = 0.19, *p* = 0.026) than the other two speeches. Electronic supplementary material, tables S17,S18 look in more detail at helplessness and empowerment.

## 5. Discussion

We analysed the effectiveness of four speeches delivered by a Global South politician to foster bonding with humanity and climate action focusing on: scientific evidence, morality, globally shared experiences and global biological kinship. The speeches were equally effective at increasing donations to a climate charity. The shared biology speech generated higher levels of fusion with humanity than the other three speeches and the two speeches that appealed to globally shared experiences and global biological kinship evoked fewer negative emotions than the speeches appealing to science and morality.

Future research might build on our results by analysing whether more comprehensive appeals to the core arguments would have more strongly divergent effects on outcomes. In the present study, the main parts of the speeches in which core arguments were experimentally manipulated consisted of only a few sentences. While the arguments based on science and morality might have appeared relatively familiar from real world debates, the arguments based on globally shared life experiences and globally shared biology were likely to have been less familiar to participants and the relevance to climate change somewhat obscure. It is possible that the full potential to affect action would require more oratorical elaboration. Future research might also explore repeated exposure to an argument and whether the corresponding effects would become weaker (e.g. via desensitization) or stronger (e.g. via reflection) over time.

Since the most effective argument to inspire climate action may differ for different individuals and social groups, having multiple arguments in the discussion might speak to the concerns of more diverse audiences than one argument or a subset of arguments alone. Thus, future research might also explore the effects of speeches that combine multiple appeals as well as the effects of exposure to multiple speeches with different arguments. Future research might also explore whether specific characteristics of the speaker influence effects by varying a single attribute of the speaker in isolation (e.g. by making two different aspects of the professional background of the same speaker salient).

A relatively consistent finding from the present study is that the two speeches appealing to shared humanity (in the form of globally shared experience or globally shared biology respectively) evoked fewer negative emotions—sadness, anger and helplessness—than the speeches appealing to science and morality. The impact of these emotions on climate relevant behaviour beyond short-term donation decisions is not yet clear. Negative emotions can be a powerful driver behind action, but they can also paralyse and limit creativity and openness towards new solutions and ambitious transformation processes. Negative emotions can also fuel political polarization over climate change, which can complicate the process of establishing a shared commitment to ambitious forms of climate action. In any case, we leave the analysis of further behavioural consequences of the emotional impacts of the speeches for future research.

In the absence of globally binding and enforceable rules, swift climate action requires a globally widespread willingness of citizens and leaders to go beyond short-term parochial interests. The search for more effective narratives to unify humanity to tackle climate change and other pressing global problems is an ongoing challenge and the future of humanity in the next decades and centuries will be profoundly affected by the answers we find.

## Data Availability

All data and code have been uploaded as part of the supplementary materials (all in Stata format). We used Stata 18.5. [38].

## Footnotes

^1^
This literature includes strategies that appeal to specific group norms (e.g. [4–6]), appeal to group image concerns (e.g. [7]) or use framing techniques such as framing climate change as a national security problem (e.g. [8]).

^2^
The global identity literature suggests that factors such as international contact [12,13], mind body practices [14] and perceptions of equality-based respect [15] might foster global identity and pro-environmental action. Loy *et al*. [16] test whether inclusive language (versus exclusive language) in the context of climate policy affects global identity and climate activism but find no significant effects.

^3^
Identity fusion has also been linked to less extreme forms of prosocial behaviour such as doing voluntary work or donating money ([20,21]).

^4^
Buhrmester *et al*. [26] demonstrate that fusion can even bridge boundaries between species by studying bonds formed with the Zimbabwean lion Cecil.

^5^
Reese & Whitehouse [31] discuss the developmental pathways of identity fusion and Whitehouse & Lanman [32] discuss the differences in cognitive foundation between identity fusion and social identification.

^6^
Links to the videos: science https://youtu.be/njH-xIKXHPM; morality: https://youtu.be/E48GVcJd8aI; shared experiences: https://youtu.be/PyLGFfbUeVA; shared biology: https://youtu.be/Yrft-WAn1MM

^7^
See As predicted no. 142506: https://aspredicted.org/GDS_MDT

^8^
Bivariate OLS regressions with robust s.e. and donations as the outcome for participants in the control condition (*n* = 540) yielded slopes of *b* = £0.32 for fusion with humanity (*p* = 0.002; 5-point scale, see electronic supplementary material, table S3), *b* = £0.24 for college education (*p* = 0.282; dummy), *b* = −£0.07 for (right-wing) political orientation (*p* = 0.180; 10-pointscale), and *b* = £0.06 for religiosity (*p* = 0.051; 10-point scale).

^9^
Effects on fusion with humanity compared with control (OLS with robust s.e.; see electronic supplementary material, table S4): science: *b* = 0.264, *p* < 0.001; morality: *b* = 0.270, *p* < 0.001; shared experiences: *b* = 0.275, *p* < 0.001; shared biology: *b* = 0.482, *p* < 0.001.

^10^
Effects on fusion with humanity compared with shared biology (OLS with robust s.e.; see electronic supplementary material, table S6): science: *b* = −0.218, *p* = 0.002; morality: *b* = −0.212, *p* = 0.002; shared experiences: *b* = −0.207, *p* = 0.003.

^11^
Effects on donations compared with control (OLS with robust s.e.; see electronic supplementary material, table S5): *b* = £0.63, *p* < 0.001; morality: *b* = £0.75, *p* < 0.001; shared experiences: *b* = £0.69, *p* < 0.001; shared biology: *b* = £0.67, *p* < 0.001.

^12^
We conducted an exploratory mediation analysis and found that fusion with humanity mediated the effect of listening to a speech versus not listening to a speech on donations (*p *< 0.001; see electronic supplementary material, table S10).

^13^
Due to spontaneous deviations from the script, there were some minor differences between the versions of the speeches (e.g. ‘incredibly similar’ (RR) versus ‘fundamentally similar’ (HW) in the shared experiences speech). We do not expect that these minor differences had any substantial effects. See electronic supplementary material, appendix §1 for scripts and videos of all speeches.

^14^
See aspredicted no. 130693 (first pilot to get feedback on earlier versions of the speeches): https://aspredicted.org/RD7_P7D, no. 136316 (main pre-registration): https://aspredicted.org/SYM_HSH and aspredicted no. 138171 (update on no. 136316): https://aspredicted.org/LFC_Q26

^15^
Note that the sample sizes for these analyses were only roughly one-third of the sample sizes of the corresponding analyses in §3 since we excluded participants from the US and South Africa.

^16^
Combining fusion with humanity and donations into an index (exploratory, not pre-registered) resulted in *p*-values for the speeches of the Western academic of 0.008 and 0.018 for the shared experiences speech and the morality speech in comparison with the control condition and 0.225 and 0.411 for the science and shared biology speech in comparison with the control condition (OLS with robust s.e.; see electronic supplementary material, table S13). Perhaps the science argument was to some degree expected from the Western academic potentially limiting its effectiveness [37] while the globally shared biology argument might have been somewhat abstract coming from a fellow British citizen.
